# Superior Inhibitory Control and Resistance to Mental Fatigue in Professional Road Cyclists

**DOI:** 10.1371/journal.pone.0159907

**Published:** 2016-07-21

**Authors:** Kristy Martin, Walter Staiano, Paolo Menaspà, Tom Hennessey, Samuele Marcora, Richard Keegan, Kevin G. Thompson, David Martin, Shona Halson, Ben Rattray

**Affiliations:** 1 University of Canberra Research Institute for Sport and Exercise, Canberra, Australia; 2 Team Danmark, Danish Elite Sport Institution, Brøndby, Denmark; 3 School of Exercise and Health Science, Edith Cowan University, Perth, Australia; 4 Endurance Research Group, School of Sport and Exercise Sciences, University of Kent, Canterbury, United Kingdom; 5 Physiology, Australian Institute of Sport, Canberra, Australia; University of Rome, ITALY

## Abstract

**Purpose:**

Given the important role of the brain in regulating endurance performance, this comparative study sought to determine whether professional road cyclists have superior inhibitory control and resistance to mental fatigue compared to recreational road cyclists.

**Methods:**

After preliminary testing and familiarization, eleven professional and nine recreational road cyclists visited the lab on two occasions to complete a modified incongruent colour-word Stroop task (a cognitive task requiring inhibitory control) for 30 min (mental exertion condition), or an easy cognitive task for 10 min (control condition) in a randomized, counterbalanced cross-over order. After each cognitive task, participants completed a 20-min time trial on a cycle ergometer. During the time trial, heart rate, blood lactate concentration, and rating of perceived exertion (RPE) were recorded.

**Results:**

The professional cyclists completed more correct responses during the Stroop task than the recreational cyclists (705±68 vs 576±74, p = 0.001). During the time trial, the recreational cyclists produced a lower mean power output in the mental exertion condition compared to the control condition (216±33 vs 226±25 W, p = 0.014). There was no difference between conditions for the professional cyclists (323±42 vs 326±35 W, p = 0.502). Heart rate, blood lactate concentration, and RPE were not significantly different between the mental exertion and control conditions in both groups.

**Conclusion:**

The professional cyclists exhibited superior performance during the Stroop task which is indicative of stronger inhibitory control than the recreational cyclists. The professional cyclists also displayed a greater resistance to the negative effects of mental fatigue as demonstrated by no significant differences in perception of effort and time trial performance between the mental exertion and control conditions. These findings suggest that inhibitory control and resistance to mental fatigue may contribute to successful road cycling performance. These psychobiological characteristics may be either genetic and/or developed through the training and lifestyle of professional road cyclists.

## Introduction

Comparisons of professional and recreational or elite and sub-elite athletes have been used to determine the factors that may contribute to successful sporting performance. With specific reference to endurance performance, several comparative studies have shown that elite athletes differ from recreational ones in a number of physiological characteristics including maximal oxygen consumption (VO_2max_), stroke volume, muscle capillary density and aerobic enzyme activity, lactate threshold and gross mechanical efficiency [1].

A limitation of this body of research is the almost exclusive examination of factors ‘below the neck’. To the best of our knowledge, the only comparative study of the brain in endurance athletes has shown increased grey matter volume in the medial temporal lobe compared to both non-exercising individuals and martial artists [2]. With regards to cognitive function, it has been recently demonstrated that faster runners during an ultramarathon outperform slower runners in terms of motor inhibition and suppression of irrelevant information, with no group differences in selective attention and working memory [3]. These findings suggest that successful endurance performance may require superior inhibitory control, a cognitive process essential for self-regulation of behaviour [4]. This proposal is plausible if we consider endurance competitions as self-regulated tasks that require the inhibition of aversive feelings (like dyspnea, muscle pain, and thermal discomfort), the urge to quit and other negative thoughts in order to reach the goal of winning or performing at the best of one’s own ability [5].

The problem with endurance competitions and other self-regulated tasks requiring inhibitory control and other effortful cognitive processes is that, over time, they can induce a state of mental fatigue or “ego depletion” [4]. Mental fatigue has been usually constructed as the negative effects of prolonged mental exertion on mood (e.g., feelings of tiredness and lack of energy) and/or performance during cognitive tasks requiring vigilance and other effortful cognitive processes [6]. However, we and others have recently demonstrated that mental fatigue is also associated with a higher perception of effort and reduced performance during physical endurance tasks [7–10]. For example, we demonstrated that performing for 30 min a cognitive task requiring strong response inhibition increases perception of effort and reduces performance in a subsequent 5K time trial on a treadmill compared to a 30-min cognitive task in which no response inhibition is required [9]. Therefore, superior resistance to mental fatigue should provide an advantage to endurance athletes. However, to date, most research on the characteristics of successful endurance athletes have focused on the physiological factors associated with superior resistance to muscle fatigue, e.g. a high percent of type I muscle fibres [1]. We are not aware of any experimental study investigating the effects of prolonged mental exertion on perception of effort and endurance performance in professional endurance athletes. This is unfortunate because a comparison between professional and recreational endurance athletes is necessary to test the hypothesis that superior resistance to mental fatigue is a psychobiological characteristic of successful endurance athletes.

The first aim of the present study was to further investigate the association between inhibitory control and endurance performance by comparing the performance of professional and recreational road cyclists in a 30-min modified incongruent colour-word Stroop task, a cognitive task requiring strong response inhibition. Based on previous findings of an association between performance level and inhibitory control in ultramarathon runners [3], we hypothesised that professional cyclists perform better in the Stroop task than their recreational counterparts. The second aim of this study was to determine whether superior resistance to mental fatigue is a psychobiological characteristic of successful endurance athletes. Because resistance to mental fatigue should provide an advantage to endurance athletes, we hypothesised that, compared to the recreational cyclists, the professionals are more resistant to the negative effects of prolonged mental exertion on perception of effort and performance during a subsequent 20-min time trial on a cycle ergometer.

## Methods

### Participants

Eleven professional, male road cyclists (23.4±6.4 years, 68.2±4.3 kg, 180±7 cm, peak power output 414±48 W, > 5 training sessions per week, > 500 km per week, > 5 years of cycling experience) and nine recreational male road cyclists (25.6±5.3 years, 80.7±11.3 kg, 177±7 cm, peak power output 261±28 W, ~3 training sessions per week, ~ 80 km per week, an average of 2 years of cycling experience) volunteered to participate in this study. Taking into account each participant peak power output and training history, and in line with guidelines designed to help describe the performance level of participants in sports science research [11], the professional cyclists were classified as performance level 5 and the recreational cyclists were classified between performance level 1 and 2. Each participant gave written informed consent prior to commencing testing. The study design and procedures were approved by the University of Canberra Committee for Ethics in Human Research. All participants received written instructions describing the study procedures but were naive to its true aims and hypotheses. Participants were led to believe the main aim of the study was to investigate the effects of mental exertion on physiological responses during the time trial. No further specifics were provided. At the end of the final visit, participants were debriefed and asked not to discuss the real aims of the study with other participants. One of the professional cyclists was unable to complete all visits due to injury. This participant’s data have been included only in the analysis of Stroop performance.

### Experimental Protocol

**Fig 1:**
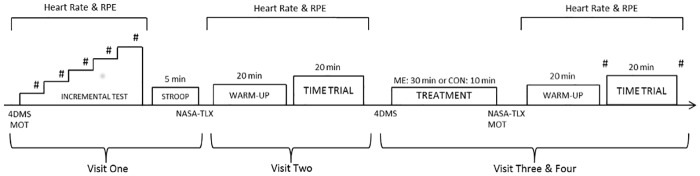
Schematic of the experimental protocol. #—Blood lactate sample. 4DMS—The Four Dimensional Mood Scale. MOT—Rating of motivation related to the time trial. NASA-TLX—The National Aeronautics and Space Administration Task Load Index. RPE—Rating of perceived exertion.

A randomised crossover design was used for the experimental component of the present study. The order of the experimental treatment (mental exertion/control or control/mental exertion) was randomly allocated based on balanced permutations generated by a web-based computer program (www.randomization.com). Participants were required to visit the laboratory on four occasions (Fig 1), in a period no longer than two weeks between the first and last visit. Testing during visits 3 and 4 was completed at the same time of the day. During the initial visit, participants completed an incremental exercise test, and became familiar with the Stroop task and all psychological, perceptual and physiological measures. During the second visit, participants were familiarised with the time trial. During visits 3 and 4, participants completed the baseline mood questionnaire, followed by either the Stroop task or the control task. After rating their motivation related to the upcoming time trial, participants were moved to a cycle ergometer where they completed a standardized warm-up and a 20 min time trial. After cooling down, participants rated again their current mood. Prior to visits three and four, participants were instructed to drink 35 ml of water per kilogram of body weight, sleep for at least 7 h, refrain from the consumption of alcohol, and avoid any vigorous exercise the day before visiting the laboratory. Participants were also instructed to avoid any caffeine and mentally demanding tasks for at least 3 h before testing. The day of visit 3, participants were asked to record the time and content of the meals consumed before testing, and to keep them consistent the day of visit 4. At the beginning of visits 3 and 4, participants were asked to complete a checklist to ascertain that they had complied with the instructions given to them. Participants were also asked to declare if they had taken any medication/drug or had an acute illness, injury, or infection on the day.

### Experimental Treatment

The mental exertion condition consisted of 30 min of modified incongruent version of the Stroop colour-word task. Participants performed this cognitive task at a computer, whilst sitting comfortably in a quiet, dimly lit room. This Stroop task consists of four words (yellow, blue, green, red) serially presented on the computer screen, displayed until the participant responded, followed by a 1.5 s rest interval. Participants were instructed to press one of four coloured buttons on the keyboard (yellow, blue, green, red), with the correct response being the button corresponding to the ink colour (either yellow, blue, green, red) of the word presented on the screen. For example, if the word blue appeared in yellow ink, the yellow button had to be pressed. If, however, the ink colour was red, the button to be pressed was the button linked to the written word, not the ink colour (e.g. if the word blue appears in red, the button blue was to be pressed). If the ink colour was blue, green or yellow, then the correct button pressed matched the ink colour. The word presented and its ink colour was randomly selected by the computer. Twenty practice attempts were allowed to ensure the participant fully understood the instructions. The Stroop task was also performed for 5 min during familiarization in visit 1. Participants were instructed to respond as quickly and accurately as possible. Visual feedback was given after each word in the form of correct or incorrect response, reaction time, and accuracy so far. Responses faster than 200 ms were excluded from the analysis as it is likely the participant responded before seeing the word [12]. Responses over 2 s were recorded as lapses and removed from the analysis. This value was chosen arbitrarily as the best trade value to normalise the data while maintaining the greatest number of responses and highest statistical power [2]. Average reaction time for the correct responses and accuracy (percentage of correct responses) were calculated for each of six 5-min epochs during the 30 min Stroop task (5^th^, 10th, 15th, 20th, 25th and 30^th^ min). The total number of correct responses were also calculated for the entire 30 min Stroop task.

The control condition consisted of an easy cognitive task performed under the same conditions as the Stroop task. Participants were instructed to sit quietly in front of the computer screen and focus for 10 min on the centred black cross, displayed on a white background.

### Incremental Exercise Test and Time Trial

During the initial visit, participants underwent an incremental exercise test to assess peak power output. The incremental exercise test was completed on a cycle ergometer (Lode Excalibur Sport, Lode, The Netherlands) with the test beginning at 125 W and increasing by 25 W every 3 min until volitional exhaustion.

Participants completed the time trial during each of the other three visits to the laboratory. A standardised warm-up was completed by all participants prior to each time trial using an SRM electromagnetically braked ergometer (High-Performance Ergometer, Schoberer Rad MeBtechnik, Germany). The time trial was then completed on another electromagnetically braked cycle ergometer (Velotron Pro, RacerMate Inc., USA). All the ergometers were fitted to replicate the participants’ bike positions. Participants were instructed to cover as much distance as possible over 20 min. The time trial began in a standard gear; however, participants were free to alter gearing throughout the time trial. A timer was placed to the front left of participants and remained visible during the time trial. Participants were blinded to all other performance and physiological data. A fan was placed behind the timer and turned on at participant’s request, and water was provided ad libitum.

During visits 3 and 4, a researcher who was blind to the experimental treatment received by the participants provided verbal encouragement throughout the test. This researcher was consistent within participants. Another researcher recorded power output at the 1^st^, 4^th^, 8^th^, 12^th^, 16^th^ and 20^th^ min of the time trial. Average speed and total distance covered during the time trial were also recorded.

### Physiological and Perceptual Measures

Capillary blood samples were collected before and straight after completion of time trial during visits 3 and 4. Samples were analysed immediately for blood lactate concentration using the Lactate Pro 2 (Arkray, Japan) analyser. During visits 3 and 4, heart rate was recorded at the end of the warm-up, and during the final 15 s of the 1^st^, 4^th^, 8^th^, 12^th^, 16^th^ and 20^th^ min of the time trial using a heart rate monitor fitted with a chest strap (T34 non-coded heart-rate transmitter, Polar, Finland).

Rating of perceived exertion (RPE) was measured using the Borg 6–20 scale [13]. During visit 1, RPE was anchored during the incremental exercise test using standard procedures [14]. During visits 3 and 4, RPE was measured at the end of the warm-up, and during the final 15 s of the 1^st^, 4^th^, 8^th^, 12^th^, 16^th^ and 20^th^ min of the time trial. At the appropriate time point, participants were asked to point on a large Borg 6–20 scale the number corresponding to their perception of effort defined as “the conscious sensation of how hard, heavy, and strenuous exercise is” [15].

### Psychological Measures

The National Aeronautics and Space Administration Task Load Index (NASA-TLX) was used to assess subjective workload of the cognitive tasks [16]. The NASA-TLX is composed of six subscales: mental demand (How much mental and perceptual activity was required?), physical demand (How much physical activity was required?), temporal demand (How much time pressure did you feel due to the rate or pace at which the task occurred?), performance (How successful do you think you were in accomplishing the goals of the task set by the experimenter?), effort (How hard did you have to work to accomplish your level of performance?) and frustration (How irritating or annoying did you perceive the task?). Participants were asked to score each of the items on a scale divided into 20 equal intervals anchored by the bipolar descriptors high and low. This score was multiplied by 5, resulting in a final score between 0 and 100 for each of the subscales. Only the mental demand, temporal demand, effort and frustration subscales were used in the present study.

The Four Dimensional Mood Scale (4DMS) was used to assess changes in mood from the beginning to the end of visits 3 and 4. The 4DMS consists of 20 adjectives and is designed to measure positive energy, tiredness, negative arousal, and relaxation. Participants rated each adjective on the extent to which it described their current mood state using a 5-point Likert scale. Reliability and validity of this scale have been previously reported [17].

Motivation related to the time trial was measured using a single item (“I am motivated to do the time trial”) scored on a 5-point Likert scale (0 = not at all, 1 = a little bit, 2 = somewhat, 3 = very much, 4 = extremely).

### Statistical Analysis

All data are presented as mean ± one standard deviation unless otherwise stated. Assumptions of statistical tests such as normal distribution and sphericity of data were checked as appropriate. Greenhouse-Geisser correction to the degrees of freedom was applied when violations to sphericity were present. Independent samples t-tests were used to determine the effect of group (professional and recreational cyclists) on the total number of correct responses during the Stroop task. A mixed 2 x 6 ANOVA was used to determine the effects of group and time (5th, 10th, 15th, 20th, 25th and 30th min) for reaction time during the Stroop task. Mixed 2 x 2 ANOVAs were used to determine the effects of group and condition (mental exertion vs. control) on the NASA TLX subscales, motivation related to the time trial, and average speed and total distance covered during the time trial. A mixed 2 x 2 x 2 ANOVA was used to determine the effects of group, condition and time (before and after the time trial) for blood lactate concentration. A mixed 2 x 2 x 2 ANOVA was used to determine the effects of group, condition and time (beginning and end of the visit) for mood. Mixed 2 x 2 x 6 ANOVAs were used to determine the effects of group, condition and time (1^st^, 4^th^, 8^th^, 12^th^, 16^th^ and 20^th^ min) on heart rate, RPE and power output during the time trial. Significant interactions were followed up with Bonferroni tests as appropriate. If significant interactions were not found, most relevant main effects are reported. Significance was set at 0.05 (2-tailed) for all analyses. The effect sizes for the repeated measures ANOVAs were calculated as partial eta squared (η²p), using the small = 0.02, medium = 0.13 and large = 0.26 interpretation for effect size [18]. All data analysis was conducted using the statistical packages for social science (SPSS version 20).

## Results

### Psychological Responses

Using the NASA-TLX, the Stroop task was rated as being more mentally demanding (grand mean mental exertion 77±11 and control 24±23, main effect of condition, p<0.001, η²p = 0.838), more temporally demanding (grand mean mental exertion 63±16 and control 12±8, main effect of condition, p<0.001, η²p = 0.887) and more frustrating (grand mean mental exertion 56±23 and control 19±19, main effect of condition, p<0.001, η²p = 0.749) than the control task, with no significant main effects of group or group x condition interactions. There was, however, a significant group x condition interaction for effort (p = 0.033, η²p = 0.240). Follow-up tests revealed that both the professional (76±19, p<0.001, η²p = 0.904) and the recreational cyclists (65±20, p = 0.001, η²p = 0.745) rated the Stroop task as more effortful than the control task, although the recreational cyclists (24±20) rated the control task as more effortful than the professional cyclists (10±19) did (p = 0.046, η²p = 0.213).

Analysis of the 4DMS revealed a decrease over time in positive energy (grand mean pre 3.0±0.7 and post 2.4±0.8, main effect of time, p<0.001, η²p = 0.361) and relaxation (grand mean pre 3.3±0.7 and post 3.0±0.8, main effect of time, p = 0.014, η²p = 0.165) with no significant main effects of group and condition, and no significant interactions. Tiredness increased over time (grand mean pre 2.2±0.5 and post 3.6±0.5, main effect of time, p<0.001, η²p = 0.771), with no significant main effects of group and condition, and no significant interactions. There were no significant main effects of time, group and condition, and no significant interactions for negative arousal (overall grand mean: 1.5±3.4).

There were no significant main effects of group and condition, and no group x condition interaction on motivation related to the time trial (overall grand mean: 2.3±0.8).

### Stroop Performance

**Fig 2:**
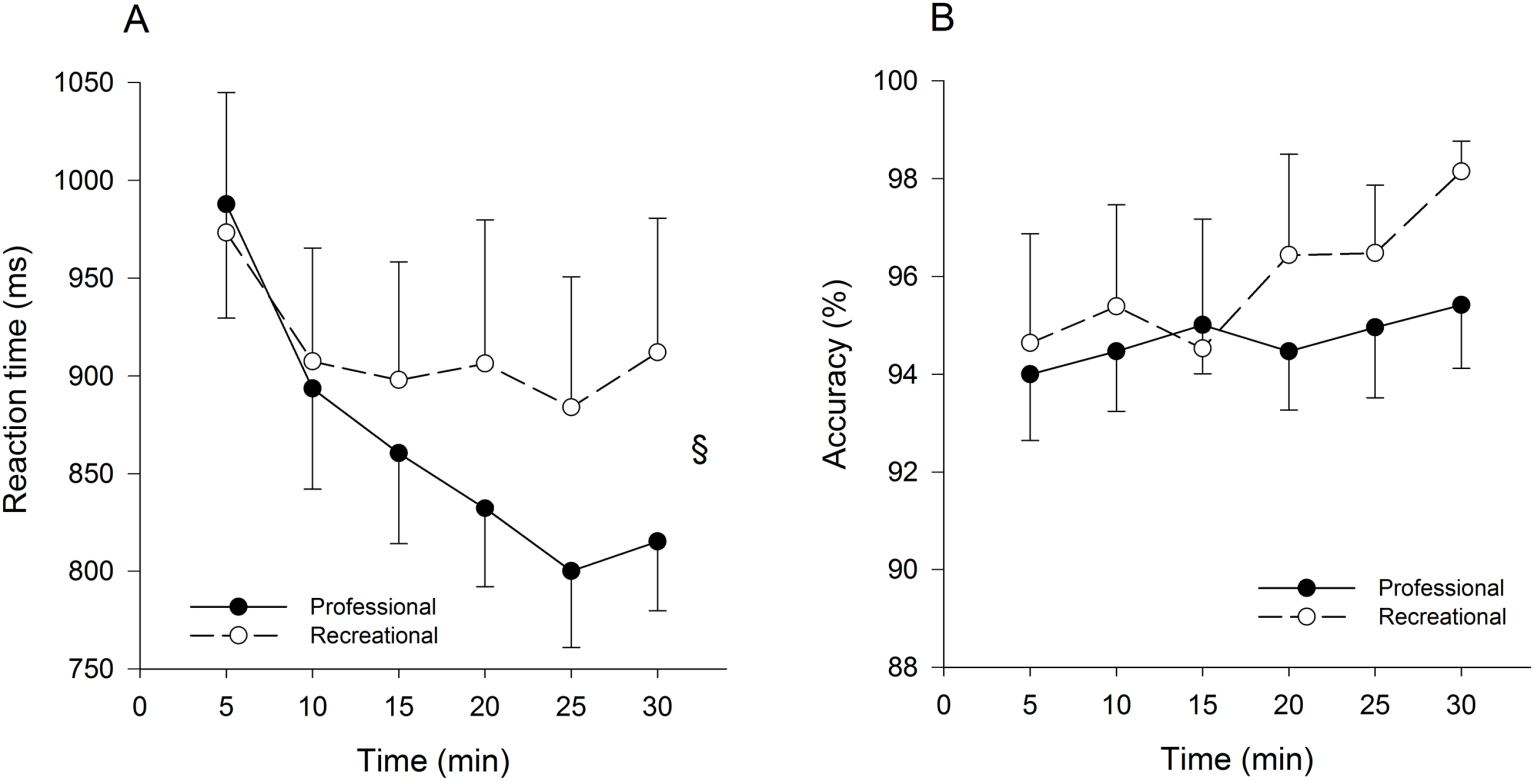
Reaction time (A) and accuracy (B) over time during the 30-min Stroop task in professional (n = 11) and recreational (n = 9) road cyclists. § Significant group x time interaction (p < 0.05). Data are presented as mean ± SEM.

**Fig 3:**
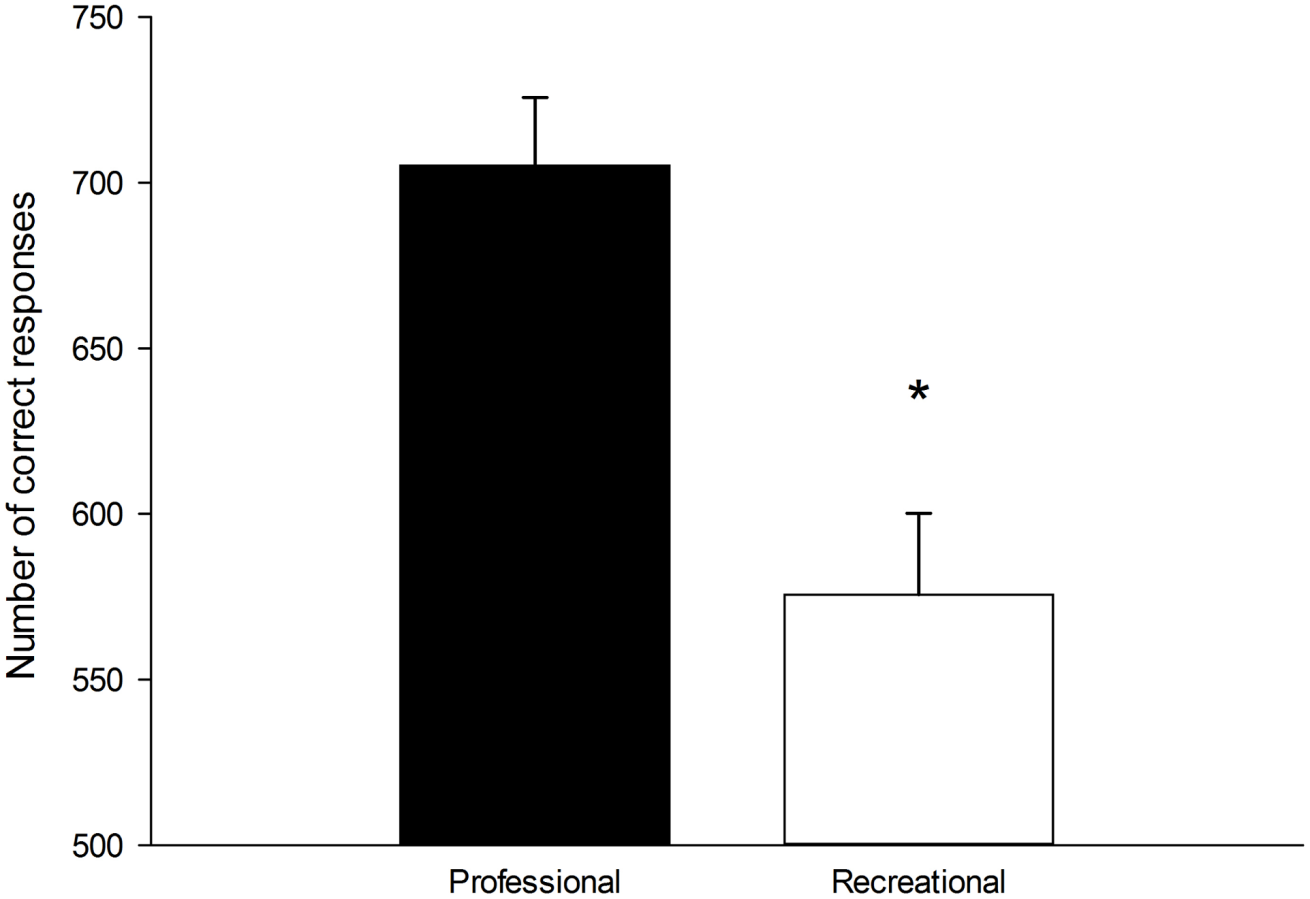
Total number of correct responses during the 30-min Stroop task in professional (n = 11) and recreational (n = 9) road cyclists. * Significant difference between groups (p < 0.05). Data are presented as mean ± SEM.

There was a significant group x time interaction for reaction time (p = 0.023, η²p = 0.165) (Fig 2a). Follow–up tests revealed that reaction time decreased over time in both the professional (p<0.001, η²p = 0.671) and recreational cyclists (p = 0.019, η²p = 0.278). However, the professional cyclists showed a greater decrease in reaction time over time compared to the recreational cyclists. There were no significant main effects of group and time, and no significant group x time interaction for accuracy (Fig 2b). In total, professional cyclists completed more correct responses than recreational cyclists (p = 0.001, η²p = 0.481) during the Stroop task (Fig 3).

### Time Trial Performance

There was a significant group x condition interaction for average speed during the time trial (p = 0.017, η²p = 0.293). Follow-up tests revealed that the professional cyclists were faster than their recreational counterparts in both the mental exertion (p<0.001, η²p = 0.822) and control condition (p<0.001, η²p = 0.857). In the professional cyclists, average speed during the time trial was not significantly different between conditions (mental exertion: 44.1±2.2 km.hr-1, control: 44.3±1.8 km.hr-1, p = 0.261, η²p = 0.138). On the contrary, the recreational cyclists were significantly slower in the mental exertion condition (34.3±2.6 km.hr-1) than in the control condition (35.5±1.9 km.hr-1, p = 0.003, η²p = 0.683).

Similarly, there was a significant group x condition interaction for total distance covered during the time trial (p = 0.019, η²p = 0.285). Follow-up tests revealed that the professional cyclists covered more distance than the recreational cyclists in both the mental exertion (p<0.001, η²p = 0.821) and control condition (p<0.001, η²p = 0.867). In the professional cyclists, total distance covered during the time trial was no significantly different between conditions (mental exertion: 14.8±0.7 km, control: 14.8±0.6 km, p = 0.223, η²p = 0.160). On the contrary, the recreational cyclists covered significantly less distance in the mental exertion condition (11.4±0.9) than in the control condition (11.8±0.6 km, p = 0.006, η²p = 0.633).

**Fig 4:**
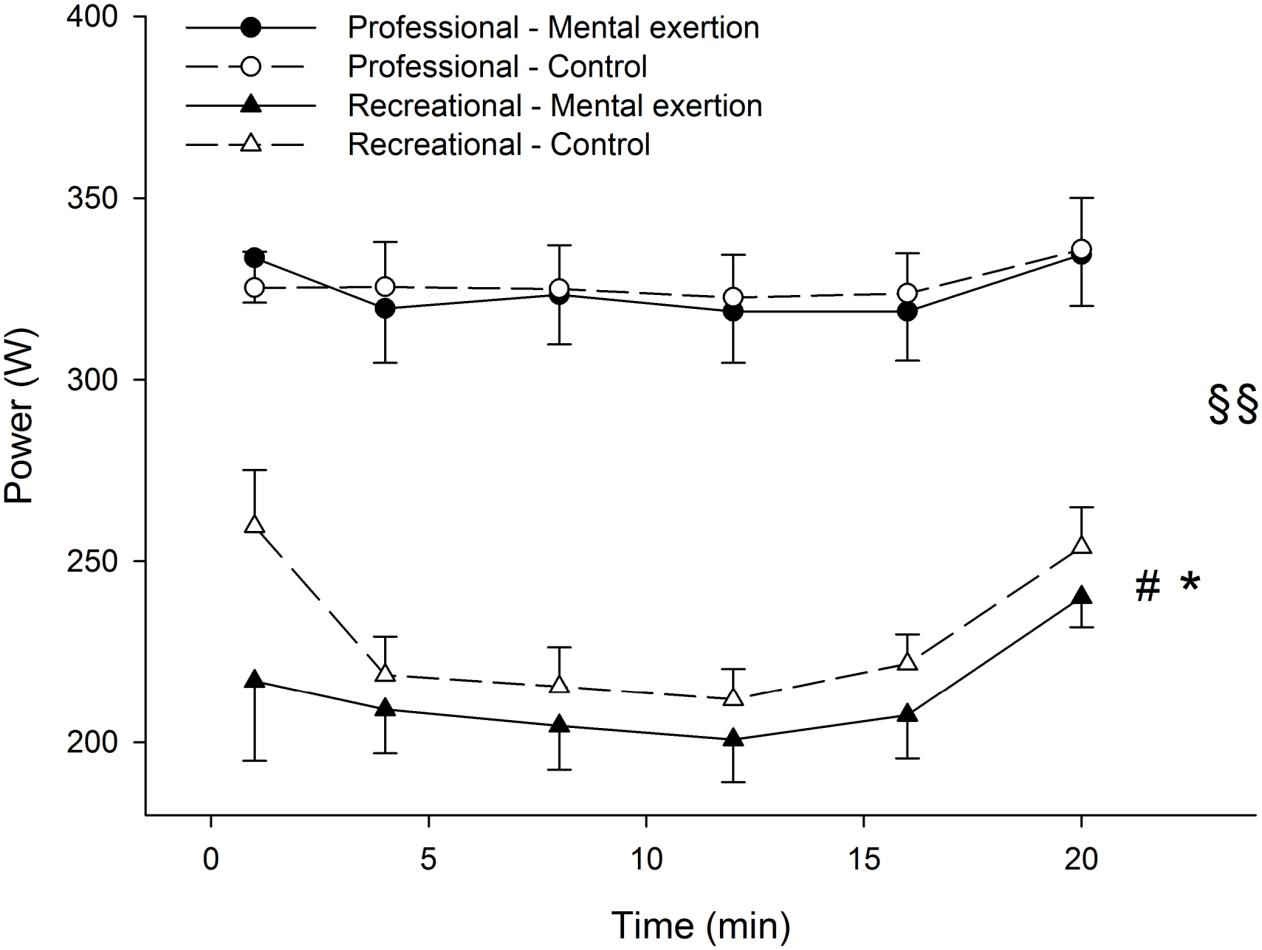
Effect of prior mental exertion on power output during the 20-min time trial in professional (n = 10) and recreational (n = 9) road cyclists. §§ Significant group x condition x time interaction (p < 0.05). # Significant main effect of time in recreational cyclists (p < 0.05). * Significant main effect of condition in recreational cyclists (p < 0.05). Data are presented as mean ± SEM.

There was a group x condition x time interaction for power output during the time trial (p = 0.049, η²p = 0.153) (Fig 4). Follow-up tests revealed that there were no significant main effects of condition (p = 0.675, η²p = 0.020) and time (p = 0.484, η²p = 0.072) in the professional cyclists. In the recreational cyclists, power output during the time trial was significantly lower in the mental exertion condition than in the control condition (main effect of condition, p = 0.017, η²p = 0.530) and increased significantly over time in both conditions (main effect of time, p = 0.003, η²p = 0.486).

### Physiological and Perceptual Responses

There were no significant interactions, and no significant main effects of group and condition for blood lactate concentration which increased significantly from before (grand mean: 3.2±1.2 mmol.l-1) to after (grand mean: 9.5±2.5 mmol.l-1) the time trial (main effect of time p<0.001, η²p = 0.846).

**Fig 5:**
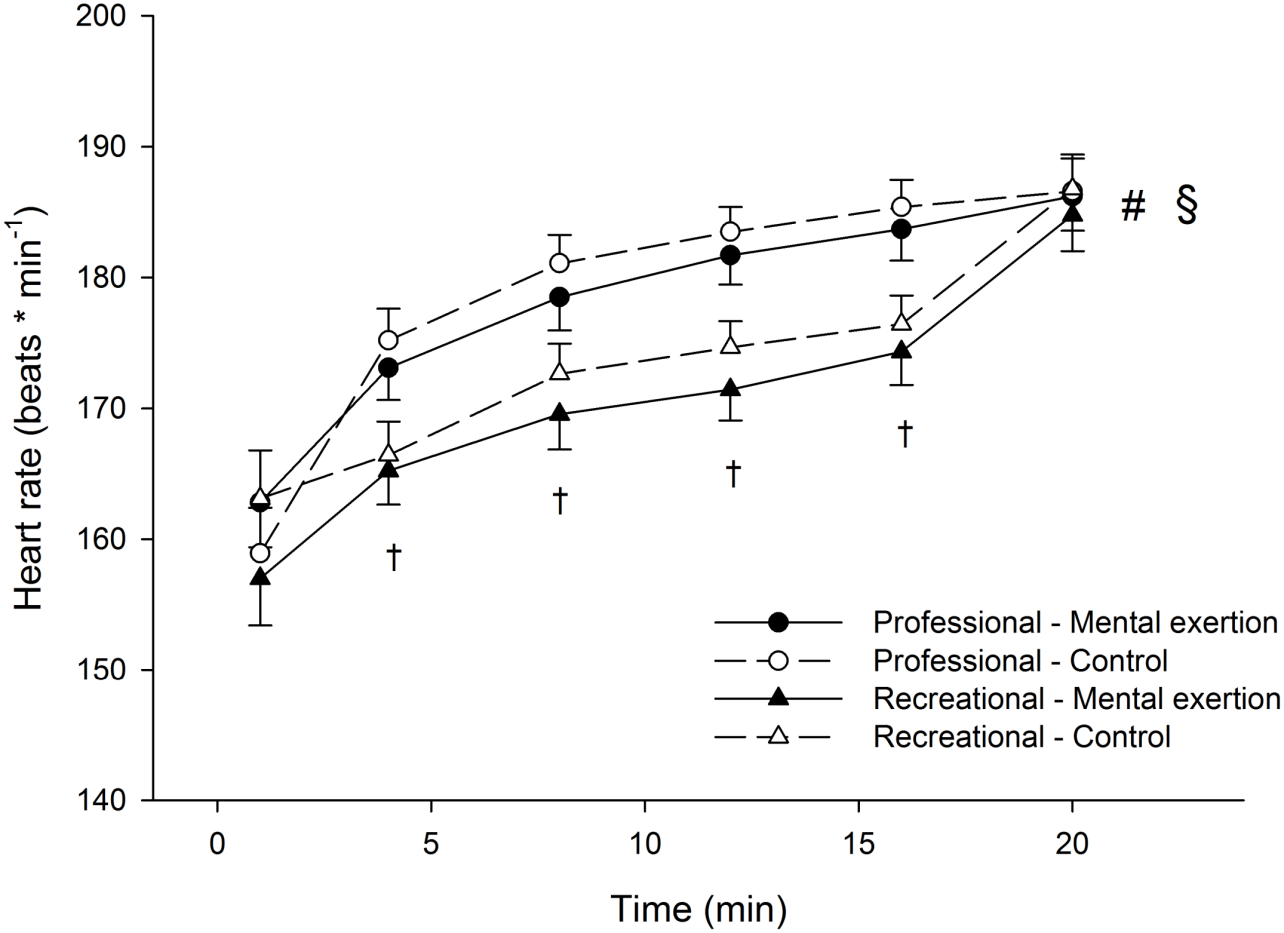
Effect of prior mental exertion on heart rate during the 20-min time trial in professional (n = 10) and recreational (n = 9) road cyclists. § Significant group x time interaction (p < 0.05). # Significant main effects of time in professional and recreational cyclists (p < 0.05). † Significant simple main effects of group (p < 0.05). Data are presented as mean ± SEM.

There were no significant group x condition x time and group x condition interactions for heart rate during the time trial (Fig 5). There was, however, a significant group x time interaction (p = 0.010, η²p = 0.234). Follow-up tests revealed that heart rate increased over time in both the professional (p<0.001, η²p = 0.773) and the recreational cyclists (p<0.001, η²p = 0.0.818). The professional cyclists had higher heart rates than the recreational cyclists at minutes 4 (p = 0.002, η²p = 0.245), 8 (p = 0.001, η²p = 0.275), 12 (p<0.001, η²p = 0.371) and 16 (p<0.001, η²p = 0.316) of the time trial.

**Fig 6:**
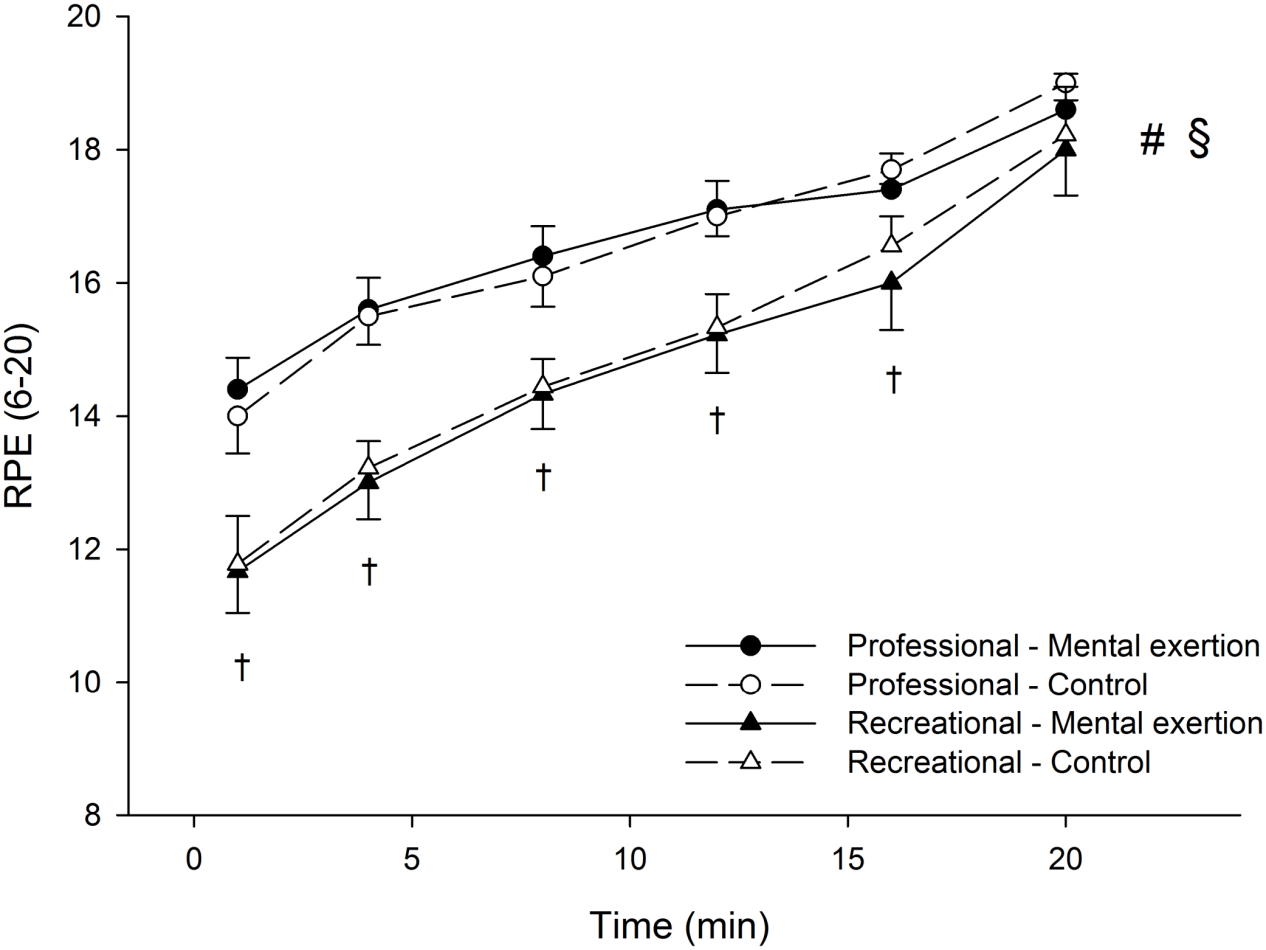
Effect of prior mental exertion on rating of perceived exertion (RPE) during the 20-min time trial in professional (n = 10) and recreational (n = 9) road cyclists. § Significant group x time interaction (p < 0.05). # Significant main effects of time in professional and recreational cyclists (p < 0.05). †Simple main effects of group (p < 0.05). Data are presented as mean ± SEM.

There were no significant group x condition x time and group x condition interactions for RPE during the time trial (Fig 6). A significant group x time interaction was found on RPE during the time trial (p = 0.005, η²p = 0.272). Follow-up tests reveal that RPE increased over time for both the professional (p<0.001, η²p = 0.748) and recreational cyclists (p<0.001, η²p = 0.895). The professional cyclists reported significantly higher RPE than recreational cyclists at minutes 1 (p<0.001, η²p = 0.338), 4 (p<0.001, η²p = 0.444), 8 (p<0.001, η²p = 0.321), 12 (p = 0.001, η²p = 0.269) and 16 (p = 0.016, η²p = 0.160) of the time trial.

## Discussion

As we hypothesised, the professional road cyclists performed better in the Stroop task than their recreational counterparts. We also found that, compared to the recreational cyclists, the professionals were more resistant to the negative effects of prolonged mental exertion on perception of effort and performance during a subsequent 20-min time trial on a cycle ergometer. These findings suggest that successful endurance performance may require superior inhibitory control and resistance to mental fatigue.

### Inhibitory control

Analysis of reaction time showed that professional cyclists progressively improved performance throughout the 30-min Stroop task whilst recreational cyclists improved their performance only over the first 10 minutes. Because accuracy was similar between the two groups, overall the professional cyclists completed significantly more correct responses than the recreational cyclists during the Stroop task. The fact that professional and recreational cyclists reported similar levels effort in relation to the Stroop task suggests that superior performance was not due to different levels of task engagement. Therefore, we propose that superior Stroop performance is indicative of better inhibitory control in professional cyclists compared to recreational ones. Our findings concur with the results of a recently published study in which the median-split technique was used to divide 30 participants into faster and slower runners based on their ranking in an ultramarathon [3]. Analysis of a battery of cognitive tests administered before their participation in the ultramarathon revealed that faster runners performed better than the slower runners in trials requiring inhibition of inappropriate motor responses, and were more effective in suppressing irrelevant information during dual-task performance. The cognitive performance of faster runners also seems to be less affected by emotional stimuli. Overall, our results and the recent findings of [3] suggest that superior inhibitory control is a psychobiological characteristic of successful endurance athletes. At an anecdotal level, this association is plausible because an endurance athlete with better inhibitory control is more likely to persist with strenuous training programs, dietary restrictions, and limitations to his/her social life while also being better able to exert control over his/her thoughts, feelings and actions during competitions.

As in other comparative studies, we can only speculate on why successful endurance athletes have superior inhibitory control. Previous studies of self-regulation suggest that inhibitory control may be a largely genetic and stable trait. Children who demonstrated greater inhibitory control by forgoing an immediate reward, for double the reward a period of time later [19] tended to have better exam scores [20], higher levels of education [21] and healthier body mass index [22] later in life than those children who chose the immediate single reward. A study of monozygotic and dizygotic twins indicated that individual differences in effortful cognitive processes including inhibitory control are almost entirely genetic in origin and largely unaffected by general intelligence or perceptual speed [23]. Genetic variation has also been associated with individual differences in brain activity related to response inhibition in Go/No-Go tasks [24]. It is, therefore, plausible that genetic factors, selected through talent identification programs and/or success in competitions, could explain the superior inhibitory control we observed in professional road cyclists.

Although inhibitory control seems to have high heritability, other research suggests that aerobic training and the lifestyle of professional athletes may also contribute. With regards to aerobic training, a structural neuroimaging study in elderly people demonstrated that 6-month aerobic training increases the volume of the anterior cingulate cortex (ACC) [25], a cortical area associated with Stroop performance [26, 27]. Furthermore, neural efficiency may also improve with aerobic training as a functional neuroimaging study showed reduced activation of the ACC during an effortful cognitive tasks in fit older adults compared to unfit individuals [28]. These cortical adaptations may mediate the specific effects of aerobic training on cognitive tasks that require inhibitory control and other effortful cognitive processes [29]. Although more neuroimaging research in young adults is required, these studies provide some support to our speculation that the high aerobic training load required by professional road cycling may be in part responsible for the superior Stroop performance we observed in the present study.

With regards to lifestyle, it is likely that professional road cyclists would encounter situations requiring self-regulation and inhibitory control on a more consistent basis than recreational ones. Professional endurance athletes must monitor their diet, alcohol intake, refrain from smoking, ensure they get enough rest and follow a strict physical training program. This consistent self-regulation of behaviour may strengthen inhibitory control across the physical and cognitive domains as demonstrated by research on self-regulatory training. For example, college students who spent 2 weeks doing one of three self-regulatory exercises (monitoring and improving posture, regulating mood, or monitoring and recording eating) performed better than a control group in a physical endurance task following a thought-suppression task [30].

### Resistance to Mental Fatigue

The second aim of this study was to test the hypothesis that professional endurance athletes have superior resistance to mental fatigue compared to their recreational counterparts. We tested this hypothesis by asking our participants to perform the Stroop task for 30 minutes and measuring the effects of this prolonged mental exertion on perception of effort and performance during a subsequent 20-min time trial on a cycle ergometer. Consistent with previous research on mental fatigue and self-paced endurance performance [7, 9], the recreational cyclists produced a lower power output for the same RPE during the time trial following the Stroop task compared to the control task. The professional cyclists, however, did not record any difference in either RPE or time trial performance between the mental exertion and control conditions. These results suggest that the professional cyclists were not mentally fatigued after performing the Stroop task for 30 minutes. We can exclude lower engagement during the Stroop task as a possible explanation for the lack of mental fatigue in the professionals. Firstly, their ratings of effort and mental demand in relation to the Stroop task were not significantly different from those of the recreational cyclists. Secondly, as discussed earlier, Stroop performance was actually better in professional road cyclists compared to their recreational counterparts. Therefore, superior resistance to mental fatigue, not lower exertion, is the most likely explanation for no negative effects of the Stroop task on perception of effort and endurance performance in professional road cyclists. Superior resistance to mental fatigue may also explain why the professionals responded more quickly than recreational cyclists in the latter stages of the Stroop task.

We are not aware of studies on the heritability of resistance to mental fatigue. However, several studies have demonstrated that the negative effects of sleep deprivation on brain activity and cognitive performance show large and stable individual differences, possibly related to adenosinergic mechanisms [31]. Given that adenosinergic mechanisms are also involved in mental fatigue [32], future research should establish whether genetic factors could explain the superior resistance to mental fatigue we observed in professional road cyclists.

With regards to environmental factors, our previous discussion on the effects of aerobic training on ACC morphology and function is relevant to superior resistance to mental fatigue in professional road cyclists. This cortical area has been associated with both mental fatigue [33, 34] and perception of effort during physical tasks [35]. Therefore, the ACC provides a plausible neurobiological link between prolonged mental exertion, high perception of effort, and reduced endurance performance [8]. Unfortunately, little is known about the effects of aerobic training on the brain of young adults. It is possible, however, that the high volume and intensity of aerobic training required by professional road cycling may induce morphological and functional adaptations in the ACC that increase resistance to mental fatigue. Further research using neuroimaging methods should test this interesting hypothesis. In addition to high training load, other psychobiological stressors (e.g., competitions, media intrusion, self-regulation of diet and other behaviours) may induce a degree of mental fatigue in a natural setting. Therefore, professional road cyclists may have been more prepared than their recreational counterparts to resist the negative effects of prolonged mental exertion on perception of effort and endurance performance.

Although our findings suggest that superior resistance to mental fatigue may be an important psychobiological characteristic of successful endurance athletes, this does not mean that successful endurance athletes are immune to mental fatigue. A limitation of the current study is that the Stroop task was quite short in duration (30 min) compared to the duration of cognitive tasks traditionally used in mental fatigue research (90 up to 180 min) [8, 36]. Furthermore, the improvement in reaction time during the Stroop task suggests that the task became progressively easier (habituation effect). Therefore, mental exertion was far from extreme in the present study. Research on overtraining syndrome clearly suggests that higher levels of psychobiological stress can induce symptoms of mental fatigue (mood disturbances, a higher-than-normal perception of effort during training, and reduced performance) even in elite athletes [37].

### Practical Applications

The results of the present study may provide a number of novel practical applications. Firstly, given the strong genetic component of inhibitory control, it is possible that the Stroop task and other cognitive tests will be used in conjunction with physiological and anthropometric tests to identify athletes that may be more likely to succeed in endurance sports. The addition of a cognitive aspect to athlete testing would extend the current talent identification process and potentially lead to a more targeted use of athlete funding. Secondly, novel interventions specifically designed to improve inhibitory control and resistance to mental fatigue may help endurance athletes looking to further enhance their performance or those struggling with self-regulation of their behaviour. Such interventions may include the teaching of self-regulatory skills as well as novel training methods in which mental exertion is combined with aerobic training [38].

## Supporting Information

S1 File Performance, physiological and psychological data. (XLSX)

S2 File Stroop performance data for professional and recreational road cyclists. (XLSX)
